# Supplementary material for: Universal non-monotonic drainage in large bare viscous bubbles
Source: Nat Commun. 2023 Feb 16;14:877. doi: 10.1038/s41467-023-36397-0 (PMC9935861; doi:10.1038/s41467-023-36397-0)
Supplement: Supplementary file 1 — Supplementary Information [file 41467_2023_36397_MOESM1_ESM.pdf]

Universal non-monotonic drainage in large bare viscous bubbles
Supplementary Information

MATERIALS AND METHODS

Method to experimentally measure the bubble film thickness

We form bubbles by injecting air from a syringe into a silicone oil bath contained by a 25 mm deep by 75 mm in diameter Petri dish. The viscosity of this silicone oils used in our experiments are μ = 150, 900, 2,300, 3,750, and 23,500 Pa·s. Using oils of such large viscosities has the advantage that film drainage can be observed over tens of minutes, whereas liquids that are orders of magnitude less viscous are expected to drain and pop in a matter of seconds or less. Indeed the drainage of the highly viscous silicone oil is so long that we can use high resolution digital time-lapse photography to capture and visualize the drainage of these viscous bubbles.

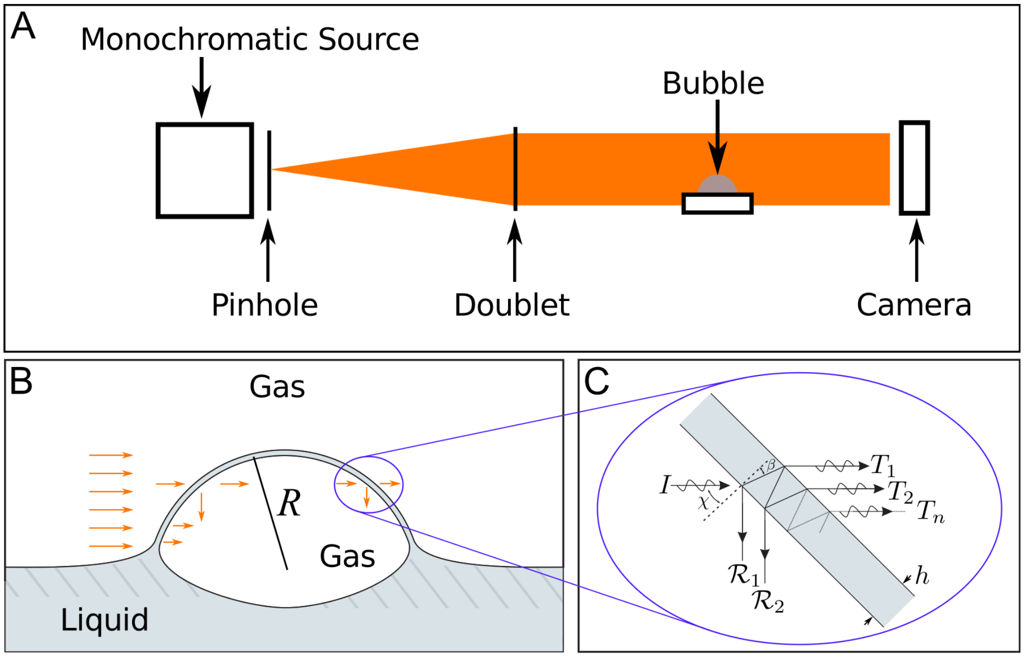

FIG. S1. Schematic of experimental setup. (A) A monochromatic light source emits light with wavelength $\lambda = 589$ nm, which passes through a pinhole and doublet before reaching the bubble. The resulting interference pattern that appears on the bubble walls is captured using a camera. (B) As the light hits the bubble, it can reflect and transmit through the bubble walls. (C) A light ray with intensity I reaches the bubble walls with thickness h at an incident angle χ. The ray is reflected with intensity $\mathcal{R}$ but also transmitted with intensity T, based on the refractive angle β.

The experimental setup used to record the bubble thickness is illustrated in Fig. S1. We place a sodium lamp emitting light at a wavelength of $\lambda = 589$ nm on an optical table behind a pinhole. The monochromatic light expands radially after passing through the pinhole before reaching a 50 mm diameter doublet at a distance equal to its focal length from the pinhole, which collimates the light from the source (Fig. S1A). The collimated beam passes through the silicone oil bubble resting on top of a silicone oil bath contained in the Petri dish. The light passes through both the front and rear faces of the bubble before being captured by a Nikon D7000 digital single lens reflex camera fitted with a f200 mm lens. Here, the front face has the surface normals oriented towards the camera, and the rear face with surface normals oriented towards the light source. As the light with intensity I and incident angle χ encounters a single face of the bubble film it multiply reflects and transmits with intensities R_1, $R_2..R_n$ and T_1, $T_2..T_n$, respectively, where n represents the number of reflections or transmissions (Fig. S1C). Because the film thickness h is small compared to the bubble radius R, the film can be assumed to be locally planar, and one can recognize the bubble film becomes a Fabry-Perot interferometer. Due to the symmetry of the film there is no effect on the relative intensity of the light transmitted through both the rear and front faces of the bubble in this Fabry-Perot interferometer. Bubble films have been recognized to replicate Fabry-Perot interferometers independently by [1] and more recently by [2, 3]. In

the projection illustrated in Fig. S1B, the incident angle χ changes with bubble arclength s. These points would correspond to a vertical line in the image taken by the camera. Meanwhile, the pixels along the edge of the captured image correspond to a great circle whose radial basis vector is orthogonal to the incoming light, or an orthodrome perpendicular to that shown in Fig. S1B. Thus for any arclength s on this great circle, the incident angle is $\chi = \pi/2$. We are unable to measure the thickness directly when $\chi = \pi/2$ because the light does not pass through the film; instead, we measure the intensity a few pixels inside this great circle on a concentric circle with radius approximately $0.995R$ (when projected on the image taken by the camera). For all points on this circle, the light strikes the bubble at an incident angle $\chi = \sin^{-1}(0.995) = 1.47$ radians.

This bubble film interferometer results in the appearance of bright and dark fringes that correspond to specific film thicknesses on the surface of the bubble . The period of "bright" fringes near the edge of the bubble film is determined by the outward pointing surface normal of the bubble $\mathbf{n}$, the angle of incidence of the light χ relative to $\mathbf{n}$, the index of refraction of the liquid film n_ℓ, the wavelength of incident light λ, and $h(s,t)$, the thickness profile of the bubble at the point s and time t. Bright fringes corresponding to constructive interference and occur at intervals of $\mathrm{n}h\cos\beta = k\lambda/2$, $k \in 1,2,3,..$, where β is angle of refraction in the liquid film that can be found through Snell's law $\sin\chi/\sin\beta = n_\ell/n_{air}$. Here $n_{air} = 1$, simplifying the expression to $\beta = \sin^{-1}(\sin\chi/n_\ell)$. "Dark" fringes corresponding to destructive interference occur at intervals $\mathrm{n}h\cos\beta = (1+2n)\lambda/4$, $k \in 0,1,2,..$ [1]. For silicone oil the index of refraction is $n_\ell = 1.4$ and the sodium lamp emits light at $\lambda = 589$ nm. The relative change in film thickness between two bright fringes observed at the edge of the bubble is $\Delta h = 278$ nm, where intensity is measured at an incident angle of $\chi = 1.47$ rad, and the corresponding angle of refraction is $\beta = 0.714$ rad.

Note that in our study, we have chosen to define $H(s)$ based on the fringes that we can directly observe. The maximum number of distinguishable fringes is nine, and thus limits the span of $H(s)$. Based on the ansatz $h(s,t) = h_0H(s)G(t)$, we define $H(s) = h(s,t)/h(0,t)$, which we then use to compute $G(t) = h(s,t)/[h_0H(s)]$. By including $H(s)$ in the calculations of $G(t)$, allows us to extend the possible range of $G(t)$ by approximately an order of magnitude larger than $H(s)$. An alternate approach would have consisted of first defining the temporal function as $G(t) = h(s,t)/h(s,0)$ and then computing $H(s) = h(s,t)/[h_0G(t)]$. This approach would have the benefit of extending the span of $H(s)$ at the expense of the span of $G(t)$. We have chosen to avoid this definition here, for one that is more traditional and direct.

Method to experimentally measure the bubble film drainage velocity

The inconsistency between the incline plug flow model and the extensional flow model motivate further experiments to test the velocity profile. We measure the film velocity using a particle tracking method. Specifically, we allow the bubbles to rise to the surface and then seed them with small particles (either talcum powder or glass microbubbles). We then simultaneously take time lapse images from the top and side to capture the position of the particles near the bubble's apex and base. Seeded particles are assumed to move with the fluid in the film, allowing us to compute the velocity as the change in positiovn of a single particle over time. The glass microbubbles were selected to have a diameter of 20 μm, which is similar to that measured for talcum powder [4]. However a key difference between the two tracers is that the talcum powder has a density larger than than silicone oil; whereas, the microbubbles have a density less than the silicone oil. Therefore in the lower potion of the bubble, where the film thickness is calculated to be larger than these particles, it would be expected that the velocity of the silicone oil would be overestimated with the talcum powder tracers and underestimated by the microbubble tracers. Near the top of the bubble, where the film is sufficiently thinner than the particle size, the mass of both talc powder and glass microbubbles is larger than the mass of the displaced film and thus could overestimate the drainage speed

Method to experimentally measure the silicone oil viscosity

We measure the viscosity of the five different silicone oils by dropping a stainless sphere (with density $\rho_p = 7,800$ $\mathrm{kg\,m^{-3}}$ and radius $R_p = 1.5$ mm) and measuring the corresponding terminal velocity u_p. The silicone oil is placed inside a cylindrical Petri dish and the sphere is then gently placed at the center to ensure that the side walls do not interfere with the velocity measurements. The speed can be theoretically estimated using Stoke's Law, where the viscous drag $6\pi\mu u_pR_p$ balances the weight of the sphere $(4/3)\pi\rho_pgR_p^3$ and the buoyancy force $(4/3)\pi\rho gR_p^3$. Based on our experimental measurement of the terminal velocity, we can thus compute the viscosity of the silicone oil bath as $\mu = (2/9)(\rho_p - \rho)gR_p^2/u_p$. The measured values of the viscosity compared to those labeled by the manufacturer are illustrated in Table I.

TABLE I. Viscosity value of the silicone oil as labeled by the manufacturer and the values measured from a falling sphere using Stoke's law.

Labeled μ (Pa·s)	Measured μ (Pa·s)
100	150
600	900
1,000	2,300
2,500	3,800
20,000	23,300

DERIVATION OF GOVERNING DRAINAGE EQUATIONS

For flow constrained to the surface of a spherical bubble, the relevant components of the rate of strain tensor $\underline{\underline{\dot{\varepsilon}}} = (\nabla \mathbf{u} + \nabla^T \mathbf{u})/2$ are:

$$\dot{\varepsilon}_{\phi\phi} = \frac{1}{R}\frac{\partial u_\phi}{\partial \phi}, \tag{1a}$$

$$\dot{\varepsilon}_{\theta\theta} = \frac{1}{R\sin\phi}\frac{\partial u_\theta}{\partial \theta} + \frac{\cot\phi}{R}u_\phi, \tag{1b}$$

$$\dot{\varepsilon}_{\phi\theta} = \frac{1}{2}\left(\frac{1}{R\sin\phi}\frac{\partial u_\phi}{\partial \theta} - \frac{\cot\phi}{R}u_\theta + \frac{1}{R}\frac{\partial u_\theta}{\partial \phi}\right). \tag{1c}$$

As demonstrated by Howell (see Eqs. (75)–(76) in [5]), the rates of strain can be related to the thickness-integrated stress tensor $\underline{\underline{\bar{\boldsymbol{\sigma}}}}$ through constitutive relations based on a Trouton model:

$$\bar{\sigma}_{\phi\phi} = 2\mu h\left(2\dot{\varepsilon}_{\phi\phi} + \dot{\varepsilon}_{\theta\theta}\right), \tag{2a}$$

$$\bar{\sigma}_{\theta\theta} = 2\mu h\left(\dot{\varepsilon}_{\phi\phi} + 2\dot{\varepsilon}_{\theta\theta}\right), \tag{2b}$$

$$\bar{\sigma}_{\phi\theta} = 2\mu h\dot{\varepsilon}_{\phi\theta}. \tag{2c}$$

Here μ is the viscosity of the fluid, h its thickness, and the overbar over the stress emphasizes that the 3D stress has been integrated over the thickness. These results also follow by noting that $p = -2\mu(\dot{\varepsilon}_{\phi\phi} + \dot{\varepsilon}_{\theta\theta})$ in the thickness-averaged Newtonian constitutive equations $\underline{\underline{\bar{\boldsymbol{\sigma}}}}/h = -p\underline{\underline{\mathbf{I}}} + 2\mu\underline{\underline{\dot{\varepsilon}}}$ that result from the incompressibility constraint $\mathrm{tr}\left(\underline{\underline{\dot{\varepsilon}}}\right) = 0$, along with $\bar{\sigma}_{rr} = 0$.

In the absence of inertial and capillary forces, conservation of momentum for thickness-integrated film can be expressed as $\nabla \cdot \underline{\underline{\bar{\boldsymbol{\sigma}}}} + \rho\mathbf{g}h = 0$. Here ρ is the film density and $\mathbf{g} = -g\hat{e}_z$ is the acceleration due to gravity. Taking the divergence of the stress in spherical coordinates, the conservation of momentum equations in the ϕ and θ directions become:

$$\frac{1}{R}\frac{\partial \bar{\sigma}_{\phi\phi}}{\partial \phi} + \frac{\cot\phi}{R}\left(\bar{\sigma}_{\phi\phi} - \bar{\sigma}_{\theta\theta}\right) + \frac{1}{R\sin\phi}\frac{\partial \bar{\sigma}_{\phi\theta}}{\partial \theta} - \rho g h\left(\hat{e}_z \cdot \hat{e}_\phi\right) = 0, \tag{3a}$$

$$\frac{1}{R\sin\phi}\frac{\partial \bar{\sigma}_{\theta\theta}}{\partial \theta} + \frac{1}{R}\frac{\partial \bar{\sigma}_{\phi\theta}}{\partial \phi} + \frac{2\cot\phi}{R}\bar{\sigma}_{\phi\theta} - \rho g h\left(\hat{e}_z \cdot \hat{e}_\theta\right) = 0, \tag{3b}$$

where $(\hat{e}_z \cdot \hat{e}_\phi) = -\sin\phi$ and $(\hat{e}_z \cdot \hat{e}_\theta) = 0$. Assuming axisymmetry ($\partial/\partial\theta \to 0$), the azimuthal balance becomes zero everywhere and the polar (ϕ-direction) balance reduces to:

$$\frac{\partial}{\partial \phi}\left(\bar{\sigma}_{\phi\phi}\sin\phi\right) - \bar{\sigma}_{\theta\theta}\cos\phi + \rho g h R\sin^2\phi = 0. \tag{4}$$

Meanwhile, conservation of mass requires $\frac{\partial h}{\partial t} + \nabla \cdot (\mathbf{u}h) = 0$. Therefore, in axisymmetric, spherical coordinates the governing equations from conservation of mass and momentum can be written as PDEs for the thickness $h(\phi, t)$ and velocity in the polar-direction $u(\phi, t) \equiv u_\phi$:

$$\frac{\partial h}{\partial t} + \frac{1}{R\sin\phi}\frac{\partial}{\partial \phi}(hu\sin\phi) = 0, \tag{5a}$$

$$2\mu\frac{\partial}{\partial \phi}\left(h\left[2\frac{\partial u}{\partial \phi}\sin\phi + u\cos\phi\right]\right) - 2\mu h\cos\phi\left[\frac{\partial u}{\partial \phi} + 2u\cot\phi\right] + \rho g R^2 h\sin^2\phi = 0 \tag{5b}$$

We proceed by seeking universal solutions for h and u that are independent of the initial conditions and can be written in the separable forms $h = h_0 H(\phi)G(T)$ and $u = (\rho g R^2/\mu)U(\phi)V(T)$. Here $T = \rho g R t/\mu$ denotes the elapsed time normalized by a visco-gravitational time scale and h_0 is a characteristic thickness defined as the thickness at the bubble apex when the time is chosen to be zero: $h_0 \equiv h(\phi = 0, T = 0)$. Applying a standard separation of variables approach, the partial differential equation describing the conservation of mass reduces to a system of two ordinary differential equations:

$$\frac{dG}{dT} + \alpha G V = 0, \tag{6a}$$

$$\frac{1}{H}\frac{dH}{d\phi} = \frac{\alpha}{U} - \frac{1}{U}\frac{dU}{d\phi} - \cot\phi. \tag{6b}$$

Here α represents a constant that arises from the separation of variables. Similarly, the conservation of momentum (force balance in the ϕ-direction) yields:

$$V(T) = 1, \tag{7a}$$

$$2\frac{\partial}{\partial\phi}\left[H\left(2\frac{dU}{d\phi}\sin\phi + U\cos\phi\right)\right] - 2H\cos\phi\left(\frac{dU}{d\phi} + 2U\cot\phi\right) + H\sin^2\phi = 0 \tag{7b}$$

Note that the standard separation of variables technique would introduce another constant to these two equations; however because V is independent of time, this constant can be absorbed into the function $U(\phi)$ such that $V(T) = 1$.

These ordinary differential equations are complemented by a set of initial and boundary conditions. By construction, $G(T = 0) = 1$ and $H(\phi = 0) = 1$, and by symmetry: $U(\phi = 0) = 0$ and $\left.\frac{dH}{d\phi}\right|_{\phi=0} = 0$. Because the highest derivative of $U(\phi)$ is second order, it requires two boundary conditions. In addition to the velocity at the bubble peak, it would be natural to select a boundary condition for the flux at the base of the bubble. Indeed, the rate at which the liquid can be absorbed into the bath controls how quickly the bubble thins and thus sets the parameter α. However, it is questionable whether the assumptions of the thin-film equations would still be appropriate at the bubble base. To overcome this challenge, we proceed by using α as a free parameter. Finally, the second boundary condition $\left.\frac{dH}{d\phi}\right|_{\phi=0} = 0$ can be converted into a second boundary condition for U by taking the limit of (6b) as $\phi \to 0$. Specifically, $\left.\frac{dU}{d\phi}\right|_{\phi=0} = \alpha/2$, which is shown in more detail in the next section.

With these conditions, the solution to (6a) is

$$G = \exp(-\alpha T). \tag{8}$$

Although the exponential function is expected from past theory and experiments, it becomes clear here that this result is not unique to a particular velocity field. Instead, drainage with exponential decay occurs whenever the drainage velocity field u is time invariant (V is constant). Our experimental results indicate that the drainage rate can be approximated as $\alpha = 0.20$ for these large bubbles. We suspect that any discrepancy in this parameter in the existing literature is largely due to uncertainties in the liquid viscosity. Indeed, relying on the reported viscosity from the distributor of the silicone oil led to a wide range in experimentally calculated α that disappeared when we independently verified the viscosity from the velocity of a sedimenting ball bearing. Even though $\alpha \approx 0.2$ in all of our experiments, it is reasonable to expect that this value would be sensitive to the precise shape and extent of the bubble cap, results that have been demonstrated experimentally and numerically [6, 7]. Therefore, we have chosen to present our theoretical results with α as a free parameter.

ANALYTIC APPROXIMATION AND NUMERIC SOLUTION TO H AND U

To obtain values for U and H requires additional effort. It is possible to decouple the equation of U from H, which simplifies the analysis. To do so, we expand the derivatives and divide by H, revealing that

$$\frac{1}{H}\frac{dH}{d\phi}\left(2\frac{dU}{d\phi}\sin\phi + U\cos\phi\right) + \frac{d}{d\phi}\left(2\frac{dU}{d\phi}\sin\phi + U\cos\phi\right) - \cos\phi\left(\frac{dU}{d\phi} + 2U\cot\phi\right) = -\frac{1}{2}\sin^2\phi, \tag{9}$$

Substituting the right-hand-side of (6b) into the first term of (7b), decouples the momentum balance so that it is solely in terms of U. By expanding the derivatives and rearranging to remove the singularity at the bubble apex, the

momentum balance can be expressed as:

$$
\begin{aligned}
2\frac{d^2U}{d\phi^2}U\sin^2\phi + 2\frac{dU}{d\phi}U\sin\phi\cos\phi - U^2\left(\sin^2\phi + 2\cos^2\phi\right) + \frac{1}{2}U\sin^3\phi + ... \\
... + \left[\alpha\sin\phi - \frac{dU}{d\phi}\sin\phi - U\cos\phi\right]\left[2\frac{dU}{d\phi}\sin\phi + U\cos\phi\right] = 0
\end{aligned}
\tag{10}
$$

Taylor approximation

We seek a Taylor expansion for the velocity profile such that $U = a_1\phi + a_2\phi^2 + a_3\phi^3 + a_4\phi^4 + O(\phi^5)$ and solve for the coefficients a_i. After expanding the cosine and sine terms as $\sin\phi = \phi - \phi^3/6 + \phi^5/120 + O(\phi^7)$ and $\cos\phi = 1 - \phi^2/2 + \phi^4/24 + O(\phi^6)$, the leading order terms of (10) simplify to:

$$3a_1(-2a_1 + \alpha)\phi^2 + O(\phi^3) = 0, \tag{11}$$

from which we can immediately find that $a_1 = 0$ or $a_1 = \alpha/2$. Because the former is a trivial solution corresponding to $U = 0$, we select the solution $a_1 = \alpha/2$. This coefficient confirms the boundary condition for the slope of U.

Continuing with the remaining terms up to $O(\phi^4)$, (10) expands to

$$3a_1(-2a_1 + \alpha)\phi^2 + (-13a_1a_2 + 5a_2\alpha)\phi^3 + \frac{1}{6}(3a_1 + 20a_1^2 - 54a_2^2 - 60a_1a_3 - 8a_1\alpha + 42a_3\alpha)\phi^4 + O(\phi^5) = 0 \tag{12}$$

Setting each order term to 0 yields the coefficients $a_2 = 0$ and $a_3 = -(3 + 2\alpha)/24$. Had we continued with the higher order terms, the even coefficients would all be 0, highlighting that U is an odd function. We can therefore approximate the spatial component of the velocity profile U as

$$U(\phi) \approx \frac{\alpha}{2}\phi - \left(\frac{3 + 2\alpha}{24}\right)\phi^3 + O(\phi^5). \tag{13}$$

Having solved for the velocity profile, we revisit (6b) to obtain an approximate solution for the thickness profile $H(\phi)$. Seeking an expansion of $H(\phi) = 1 + b_1\phi + b_2\phi^2 + b_3\phi^3 + O(\phi^4)$, which satisfies the boundary conditions, the same procedure can be followed to determine the coefficients b_i. Using the Taylor expansion for cosine and sine, (6b) simplifies to

$$\frac{1}{2}b_1\alpha\phi^2 + O(\phi^3) = 0, \tag{14}$$

from which we immediately deduce that $b_1 = 0$. Using the coefficients of the velocity profile, we can determine the remaining leading order terms by expressing (6b) as:

$$\frac{1}{2}b_1\alpha\phi^2 + \left(-\frac{1}{2} - \frac{\alpha}{2} + b_2\alpha\right)\phi^3 + \frac{1}{24}(-15b_1 - 16b_1\alpha + 36b_3\alpha)\phi^4 + O(\phi^5) = 0. \tag{15}$$

This analysis highlights $H(\phi)$ as an even function, as $b_2 = (1 + \alpha)/2\alpha$ and $b_3 = 0$, leading to the following Taylor expansion for the spatial thickness profile:

$$H(\phi) \approx 1 + \left(\frac{1 + \alpha}{2\alpha}\right)\phi^2 + O(\phi^4). \tag{16}$$

Padé approximation

Using the results from the Taylor expansions, we can also derive a Padé aproximant. Starting with the velocity, we can approximate its spatial profile using a function of the form $U(\phi) = (A_0 + A_1\phi)/(1 + B_1\phi + B_2\phi^2)$, which after linearizing simplifies to:

$$U(\phi) \approx A_0 + (A_1 - A_0B_1)\phi + (-A_1B_1 + A_0B_1^2 - A_0B_2)\phi^2 + (A_1B_1^2 - A_0B_1^3 - A_1B_22A_0B_1B_2)\phi^3 + O(\phi^4) \tag{17}$$

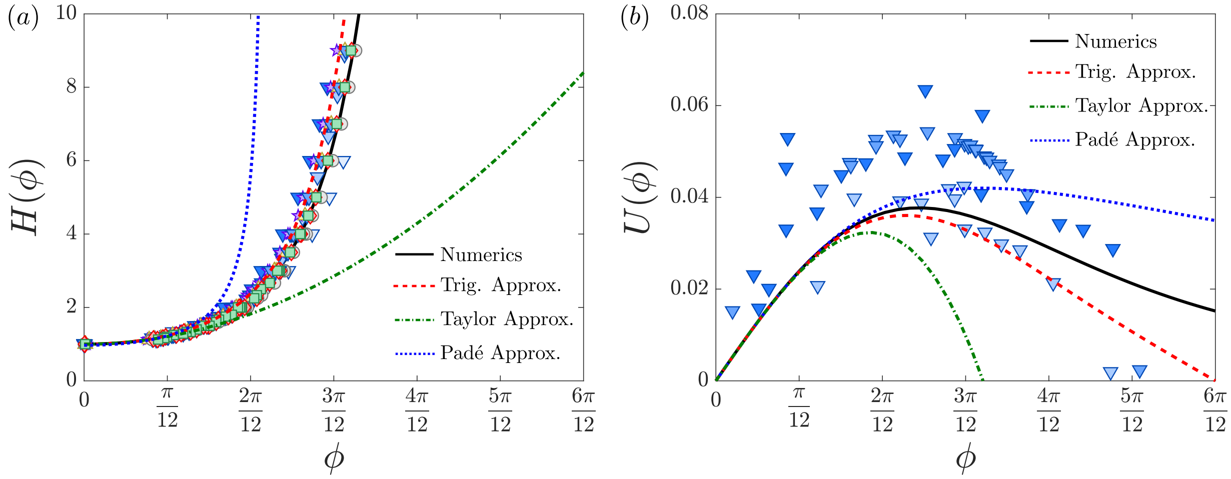

FIG. S2. Comparison between experimental results (symbols) and theory (lines). (a) The thickness spatial profile $H(\phi)$ increases monotonically the polar angle ϕ, with the numerical solution (solid line) accurately capturing the trend of the experimental data. Even though the Taylor (dot-dash line) and Padé (dotted line) approximations fail to describe the profile beyond small angles, the trigonometric approximation (dashed line) agrees well with our experimental results. (b) The velocity profile increases non-monotonically with the polar angle ϕ, exhibiting a maximum near 0.6 rad. Both the numerics and trigonometric approximations follow the trend of the experimental data; whereas the Taylor and Padé (dotted line) approximations again deviate beyond small angles.

Matching each order term with the ones in (13), we find that $A_0 = 0$, $A_1 = \alpha/2$, $B_1 = 0$, and $B_2 = (3 + 2\alpha)/12\alpha$, leading to the following for the velocity

$$U(\phi) \approx \frac{\frac{\alpha}{2}\phi}{1 + \left(\frac{3+2\alpha}{12\alpha}\right)\phi^2}. \tag{18}$$

Using a similar analysis by matching the boundary condition and curvature at the origin, we can derive a Padé approximant for the thickness profile, which leads to:

$$H(\phi) \approx \frac{1}{1 - \left(\frac{1+\alpha}{2\alpha}\right)\phi^2} \tag{19}$$

The Taylor and Padé approximations for the thickness and velocity are illustrated in Fig. S2. Both approximations match the numerical solution (solid line) well at early values of ϕ but start to deviate beyond $\phi > 2\pi/12$. The Taylor expansion underestimates the thickness and speed; whereas the Padé approximant overestimates the results. However, both approximations fail to provide a reasonable value for the speed near the bubble's edge ($\phi = \pi/2$), where we would expect a minimal speed near the stationary meniscus. Because the speed is an odd function and the thickness even, a zero speed at the meniscus would result in a divergence in the thickness.

Trigonometric approximation

Motivated by the Padé approximant and a divergence of the thickness near the meniscus, we seek an *ad hoc* trigonometric approximation for the thickness of the form $H(\phi) = \cos^{-m}\phi$, where we force a pole at $\pi/2$. Such an approximation is quite similar to the result of Debregeas et al., who assumed an inclined plug flow to get a thickness of the form $H(\phi) = \cos^{-4}(\phi/2)$. While this result does not agree with our experimental data, a different power of m might lead to a better approximation. After linearizing the trigonometric approximation and matching the curvature, we find that $m = (1 + 1/\alpha)$. We can therefore derive functions for the thickness profile, and by extension the velocity profile using (6b), such that:

$$H(\phi) \approx \frac{1}{\cos^{\left(1+\frac{1}{\alpha}\right)}\phi} \tag{20}$$

$$U(\phi) \approx \alpha^2 \left(\frac{\cos\phi - \cos^{\left(1+\frac{1}{\alpha}\right)}\phi}{\sin\phi}\right) \tag{21}$$

Numeric solution to H and U

The values of U and H are numerically solved with an explicit Runge-Kutta algorithm. Equation (10) is recast by isolating the second derivative of U on one side of the equation. Yet doing so, introduces a singularity at the bubble apex ($\phi = 0$), which is where both boundary conditions originate. Therefore the simulations are initiated at a small angle $\phi_0 = 10^{-5}$ rad from the apex with $U(\phi_0) = 0$, $\left.\frac{dU}{d\phi}\right|_{\phi=\phi_0} = \frac{\alpha}{2}$, and $\alpha = 0.2$. Once numerical values $U(\phi)$ are obtained, they can be substituted into (6b) so that H can be explicitly solved with the boundary condition $H(0) = 1$. The numerical results (solid line) agree with the experimental data quite well for both the thickness and the velocity (Fig. S2).

SUPPLEMENTARY REFERENCES

[1] H. Princen and S. Mason, Optical interference in curved soap films, J. Colloid Sci. **20**, 453 (1965).

[2] Y. Couder, E. Fort, C.-H. Gautier, and A. Boudaoud, From bouncing to floating: noncoalescence of drops on a fluid bath, Phys. Rev. Lett. **94**, 177801 (2005).

[3] F. Müller, U. Kornek, and R. Stannarius, Experimental study of the bursting of inviscid bubbles, Physical Review E **75**, 065302 (2007).

[4] C. R. Gilbert, B. R. Furman, D. J. Feller-Kopman, and P. Haouzi, Description of particle size, distribution, and behavior of talc preparations commercially available within the united states, Journal of bronchology & interventional pulmonology **25**, 25 (2018).

[5] P. Howell, Models for thin viscous sheets, Eur. J. Appl. Maths **7**, 321 (1996).

[6] F. Pigeonneau and A. Sellier, Low-reynolds-number gravity-driven migration and deformation of bubbles near a free surface, Physics of Fluids **23**, 092102 (2011).

[7] H. Kočárková, F. Rouyer, and F. Pigeonneau, Film drainage of viscous liquid on top of bare bubble: Influence of the bond number, Physics of Fluids **25**, 022105 (2013).
