# Supplementary material for: Universal non-monotonic drainage in large bare viscous bubbles
Source: Nat Commun. 2023 Feb 16;14:877. doi: 10.1038/s41467-023-36397-0 (PMC9935861; doi:10.1038/s41467-023-36397-0)
Supplement: Supplementary file 3 — Description of Additional Supplementary Files [file 41467_2023_36397_MOESM3_ESM.pdf]

Description of Additional Supplementary Files

File Name: Supplementary Movie 1
Description: Timelapse images of the viscous bubble backlit with a sodium lamp reveal interference patterns that are used to calculate the film thickness in time and space.

File Name: Supplementary Movie 2
Description: Small particles are seeded in the film and used as tracers to estimate the drainage velocity.
